# Bivalirudin vs. heparin on a background of ticagrelor and aspirin in patients with ST-segment elevation myocardial infarction undergoing primary percutaneous coronary intervention: A multicenter prospective cohort study

**DOI:** 10.3389/fcvm.2022.932054

**Published:** 2022-10-28

**Authors:** Xiao-Fan Yu, Hong-Wu Chen, Jie Xu, Qi-Zhi Xu, Xiao-Hong Zhang, Bin-Bin Li, Bang-Long Xu, Li-Kun Ma

**Affiliations:** ^1^Division of Life Sciences and Medicine, Department of Cardiology, The First Affiliated Hospital of USTC, University of Science and Technology of China, Hefei, China; ^2^Department of Cardiology, The First People's Hospital of Hefei City, Hefei, China; ^3^Department of Cardiology, The Second Affiliated Hospital of Anhui Medical University, Hefei, China

**Keywords:** bivalirudin, heparin, ticagrelor, ST-segment elevation myocardial infarction, primary percutaneous coronary intervention

## Abstract

**Objective:**

Current guidelines recommend potent P2Y12 inhibitors such as ticagrelor over clopidogrel as part of the dual antiplatelet therapy (DAPT) after ST-segment elevation myocardial infarction (STEMI), irrespective of final management strategy. The aim of this multicenter prospective cohort study was to examine the efficacy and safety of bivalirudin with background ticagrelor and aspirin therapy in patients with STEMI undergoing primary percutaneous coronary intervention (PPCI).

**Methods:**

A total of 800 patients with STEMI who were undergoing PPCI and receiving treatment with aspirin and ticagrelor from three Hospitals between April 2019 and September 2021 were included in this study. The patients were assigned, according to the perioperative anticoagulant, to the bivalirudin group (*n* = 456) or the heparin group (*n* = 344). In this study, the primary endpoint was 30-day net adverse clinical events (NACEs), a composite of major adverse cardiac or cerebral events (MACCEs, a composite of cardiac death, recurrent myocardial infarction, ischemia-driven target vessel revascularization, or stroke), or any bleeding as defined by the Bleeding Academic Research Consortium (BARC) definition (grades 1–5).

**Results:**

The patients were followed up for 30 days after PPCI. The incidence of NACE was significantly lower in the bivalirudin group than in the heparin group (11.2 vs. 16.0%, *P* = 0.042), and this significance was mainly a consequence of the reduction in BARC 1 bleeding events in the bivalirudin group compared to the heparin group (3.2 vs. 7.1%, *P* = 0.010). Results from multivariate Cox regression analysis showed that bivalirudin significantly reduced 30-day NACE (HR: 0.676, 95% CI: 0.462–0.990, *P* = 0.042) and BARC1 bleeding events (HR: 0.429, 95% CI: 0.222–0.830, *P* = 0.010). No significant between-group differences were observed for MACCE, all-cause mortality, cardiac death, recurrent myocardial infarction, stroke, target vessel revascularization, stent thrombosis, and BARC2-5 bleeding events at 30 days.

**Conclusion:**

In patients with STEMI who were undergoing primary PCI and receiving treatment with aspirin and ticagrelor, bivalirudin was associated with decreased rates in NACE and minimal bleeding events without significant differences in the rates of MACCE or stent thrombosis when compared with heparin. Nevertheless, large randomized trials are warranted to confirm these observations.

**Clinical trial registration:**

The trial was registered at the Chinese Clinical Trial Registry (ChiCTR, http://www.chictr.org.cn; identifier [ChiCTR1900022529]). Registered on 15 April 2019. Registration title: Effect of bivalirudin combined with ticagrelor in patients with ST-segment elevation myocardial infarction during primary percutaneous coronary intervention.

## Introduction

The prognosis for patients with ST-segment elevation myocardial infarction (STEMI) is improved with the use of primary percutaneous coronary intervention (PPCI). Anticoagulation with bivalirudin or heparin, in combination with antiplatelet agents such as aspirin, P2Y12 inhibitors, and glycoprotein IIb/IIIa inhibitors, is essential to prevent adverse ischemic events, especially stent thrombosis and reinfarction during and after primary PCI in patients with STEMI (1).

Current guidelines recommend potent P2Y12 inhibitors such as ticagrelor over clopidogrel as part of the dual antiplatelet therapy (DAPT) after STEMI (because prasugrel is not listed in China, ticagrelor was selected as potent P2Y12 inhibitors in this study), irrespective of final management strategy (2, 3). In the PLATO trial (4), ticagrelor significantly reduced the rate of composite ischemic endpoints after STEMI compared with clopidogrel. However, the ischemic benefit came with an increased risk of bleeding, which has been shown to adversely affect prognosis (5–7). Choosing the best procedural anticoagulation regimen to balance the risks of ischemia and bleeding during primary PCI is essential to optimize outcomes (8).

Bivalirudin and heparin are the two anticoagulant drugs most commonly used during PCI (9). Several trials reported a reduced bleeding risk with bivalirudin vs. heparin with or without glycoprotein IIb/IIIa inhibition in patients undergoing primary PCI for STEMI (10–12). Bivalirudin may mitigate any increase in bleeding seen with ticagrelor (13). This suggests that bivalirudin may be a better choice of anticoagulant than heparin in patients with STEMI treated with ticagrelor in this setting. However, the efficacy and safety of bivalirudin combined with ticagrelor during primary PCI have not been established in patients with STEMI (8). Therefore, this multicenter prospective cohort study was designed to compare the perioperative efficacy and safety of bivalirudin and heparin on the basis of antiplatelet therapy of ticagrelor combined with aspirin in patients with STEMI undergoing primary PCI.

## Materials and methods

### Subjects

**Figure 1:**
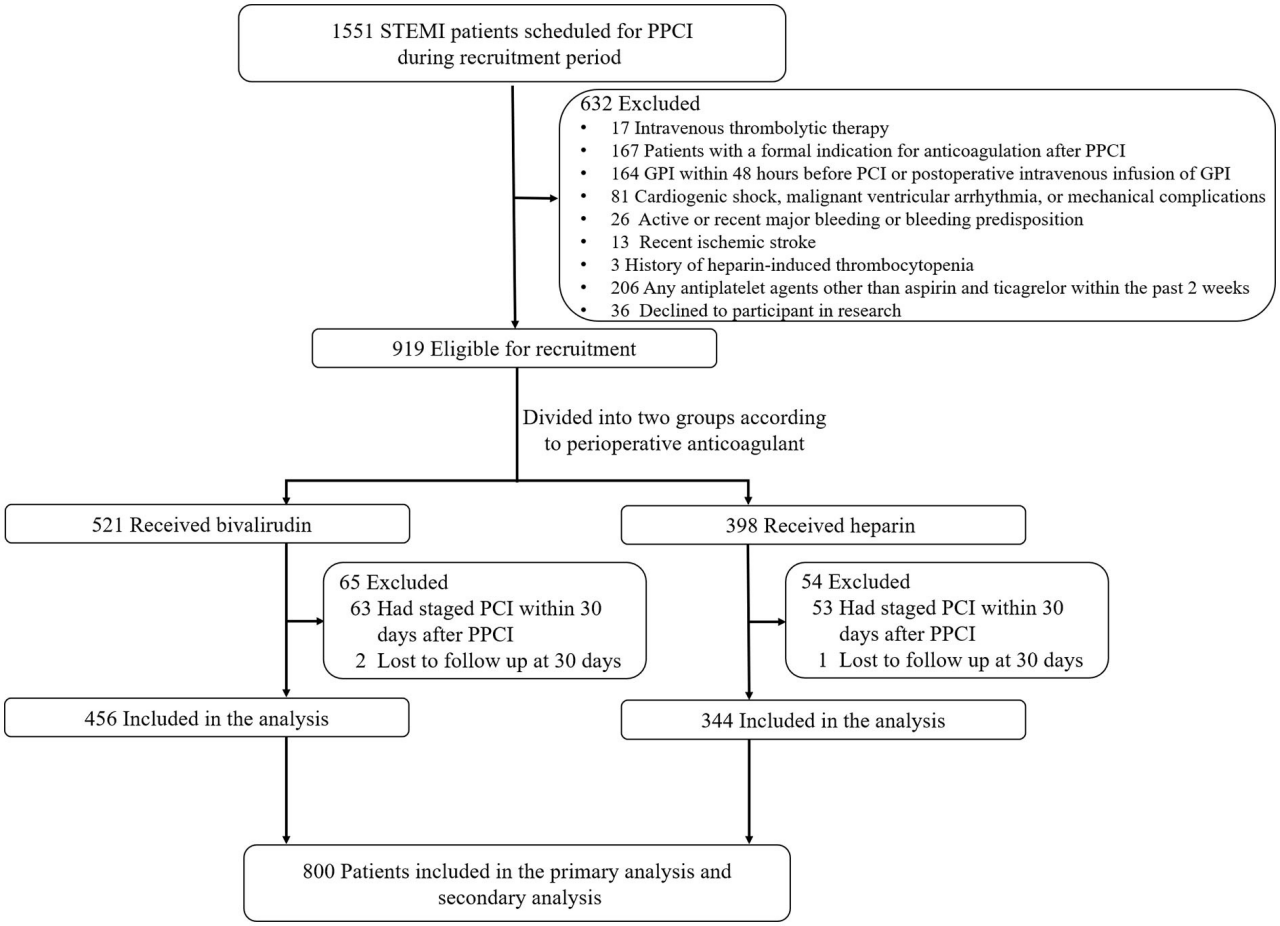
Flow diagram of the study. PPCI, primary percutaneous coronary intervention; GPI, glycoprotein IIb/IIIa inhibitors.

A total of 800 patients with STEMI who were undergoing PPCI and receiving treatment with aspirin and ticagrelor from three hospitals (The First Affiliated Hospital of the University of Science and Technology of China, The First People's Hospital of Hefei City, and The Second Affiliated Hospital of Anhui Medical University) in China between April 2019 and September 2021 were included in this study (Figure 1).

### Enrollment criteria

Inclusion criteria: (1) use of antiplatelet agents (aspirin concomitant with ticagrelor, loading or maintenance dose) before PPCI; (2) STEMI with PPCI of culprit lesion; (3) no revascularization for other non-culprit target vessels within 30 days after PPCI; (4) ability to understand and to comply with the study protocol; and (5) signed informed consent form.

Exclusion criteria: (1) intravenous thrombolytic therapy; (2) patients with a formal indication for anticoagulation after PPCI (e.g., atrial fibrillation, left ventricular thrombus, intra-aortic balloon pump, pulmonary embolism, mechanical heart valve); (3) glycoprotein IIb/IIIa inhibitor (GPI) within 48 h before PCI or postoperative intravenous infusion of GPI; (4) cardiogenic shock, malignant ventricular arrhythmia, or mechanical complications; (5) severe hematologic disease or history of intracerebral mass, aneurysm, arteriovenous malformation, recent (<6 months) ischemic stroke, recent (<6 months) intracranial hemorrhage or, gastrointestinal or genitourinary bleeding within the past 2 weeks; (6) history of heparin-induced thrombocytopenia; (7) suspected acute aortic dissection (AAD); (8) known allergy to any study drug; (9) patients with any indication for chronic anticoagulation; (10) bivalirudin and heparin were both used during the peri-procedural period of PCI; (11) any antiplatelet agents other than aspirin and ticagrelor within the past 2 weeks; and (12) current participation in an investigational drug or device trial. GPI use before catheterization or intended use during PCI was contraindicated; however, bail-out use of GPI was allowed and recorded.

This study was approved by the ethics committee at the First Affiliated Hospital of the University of Science and Technology in China (2019-ky03, Supplementary Figure 1). Oral informed consent was obtained from all participants before recruitment, and written informed consent was obtained within 24 h after recruitment.

### Clinical data collection

For eligible subjects, the information on baseline characteristics (gender, age, coronary risk factors, laboratory tests), clinical medications, and detailed PPCI data were collected by designated study staff. A follow-up telephone contact or office visit 30 days after the emergency PCI was conducted for all subjects. The primary endpoint was 30-day net adverse clinical events (NACEs), a composite of major adverse cardiac or cerebral events (MACCEs, a composite of cardiac death, recurrent myocardial infarction, ischemia-driven target vessel revascularization, or stroke) or any bleeding as defined by the Bleeding Academic Research Consortium (BARC) (14) definition (grades 1–5). Major secondary endpoints were MACCE, any bleeding, and certain or probable stent thrombosis at 30 days as defined by the Academic Research Consortium (ARC) (15).

### Medical treatment

Subjects took a single dose of aspirin (100–300 mg) and ticagrelor (180 mg) before PPCI. Interventional procedures were performed according to standard techniques and the choice between bivalirudin and heparin was at the operators' discretion. Bivalirudin (250 mg/vial, Salubris Pharmaceutical Co, Shenzhen, China) could be started immediately as a bolus of 0.75 mg/kg followed immediately by an infusion of 1.75 mg/kg/h during angiography, and continued during PPCI. ACT was recommended monitoring at 5 min after the first bolus of bivalirudin. If the ACT is <225 seconds, an additional bolus of 0.3 mg/kg was given. The sheath has been removed usually at the end of the procedure. At 30 min after the end of PCI, the physicians decided whether or not to administer bivalirudin (1.75 mg/kg, iv) as needed for no more than 4 h (from the start of this dose). Patients in the heparin group were given a loading dose (100 U/kg) of heparin. ACT was monitored 5 min after the initial dose, and a heparin 20 U/kg bolus was given if ACT was <225 s. After PPCI, the patients were instructed to take aspirin (100 mg, qd) and ticagrelor (90 mg, bid). The use of aspirin lifelong was advised and ticagrelor was prescribed for 12 months. Unless contraindicated, long-term lipid-lowering therapy was recommended. The use of other medications (e.g., beta-blockers, angiotensin-converting enzyme inhibitors, or angiotensin II receptor blockers) was left to the discretion of the treating physicians.

### Definitions

Hypertension was defined by an SBP >140 mmHg or a DBP >90 mmHg. Anemia was defined as hemoglobin <120 g/L for men or hemoglobin <110 g/L for women. Cardiac death was defined as any death with a clear relationship to cardiac factors (e.g., myocardial infarction, heart failure, fatal arrhythmia), death of unknown cause, and all procedure-related death, including concurrent treatment-related death. The definition of myocardial infarction was based on the European Society of Cardiology (ESC)/American College of Cardiology (ACC)/American Heart Association (AHA)/World Heart Federation (WHF) Task Force for the Fourth universal definition of myocardial infarction criteria (16). Ischemia-driven target vessel revascularization (TVR) was defined as second PCI or coronary artery bypass grafting (CABG) due to re-stenosis of the target lesion or any part of the same major vessel. Stroke was defined as the presence of a new focal neurologic deficit thought to be vascular in origin, with signs or symptoms lasting more than 24 h or 24 h because of pharmacologic or non-pharmacologic intervention. The preoperative estimated glomerular filtration rate (eGFR) was calculated from serum creatinine (sCr) concentrations using the modified glomerular filtration rate estimating equation for Chinese population: eGFR (ml/min/1.73 m^2^) = 175 × (sCr)-1.234 × (age)-0.179 × (0.79 if patient is a female subject) (17).

### Sample size and statistical analysis

A multivariable Cox regression model was used to analyze the effect of anticoagulants (bivalirudin vs. heparin) on the primary endpoint (NACE). According to the BRIGHT study (12), the estimated incidence of NACE at 30 days of follow-up was 10%. The variable of interest was X1 (perioperative anticoagulant, i.e., bivalirudin vs. heparin), and the estimated log hazard ratio lnΔ = 0.53. With a two-sided significance level of 0.05 and statistical power of 80%, the calculation process of the required sample size is as follows: ① estimate the standard deviation of X1 and obtain σ = 0.6; ② perform multiple linear regression analysis on X1 and other covariates and obtain *R*^2^ = 0.0137. Using the PASS 11 software (Supplementary Figure 2), the above parameters were included in the calculation, and the sample size was 787 subjects (≈800 total subjects).

Continuous variables were demonstrated as mean ± standard deviation (SD) or median value [interquartile range (IQR)]. Frequency and proportion were presented for categorical variables. For group comparisons, Pearson's chi-square test or Fisher's exact test was used for categorical variables when appropriate. Student's unpaired *t*-test or the Mann–Whitney rank-sum test was used for continuous variables when appropriate. For each primary and secondary endpoint, Kaplan–Meier methods were used to estimate 30-day event rates for each group, and comparisons between the two study groups were performed using the log-rank test. A multivariable Cox regression model for clinical endpoints was performed, correcting for those clinical variables significantly different between groups, with a final adjustment model including age, gender, smoking status, eGFR, previous myocardial infarction, and anemia. SPSS v24.0 was used for statistical analysis. All tests were two-sided, and *P* < 0.05 was considered to be statistically significant.

## Results

### Clinical characteristics

As shown in Tables 1, 2, the bivalirudin group was older, with a higher prevalence of anemia and lower prevalence of male subjects, prior or current smoking, and previous MI than the heparin group. There were no significant differences in perioperative procedures and medications between the two groups. GPIs were administered in 11.8 and 12.4% of patients in the bivalirudin and UFH groups, respectively. In the bivalirudin group, all patients received a post-procedure infusion of the 1.75 mg/kg/h bivalirudin PCI dose for a median duration of 180 min (IQR, 125–240 min).

**Table 1:** Basic clinical characteristics.

**Variable**	**Bivalirudin group (*n =* 456)**	**Heparin group (*n =* 344)**	***P*-value**
Age (years)	66.7 ± 12.7	62.0 ± 13.7	<0.001
Men [*n* (%)]	326 (71.5)	270 (78.5)	0.025
BMI, kg/m^2^	23.9 ± 3.3	24.6 ± 3.4	0.112
Heart rate	78.9 ± 18.2	80.1 ± 15.7	0.326
LVEF (%)	54.6 ± 10.9	55.7 ± 11.3	0.305
Prior or current smoking [*n* (%)]	139 (30.5)	141 (41.0)	0.002
Hypertension, [*n* (%)]	276 (60.5)	193 (56.1)	0.209
Hyperlipidemia [*n* (%)]	89 (19.5)	63 (18.3)	0.667
Previous stroke [*n* (%)]	36 (7.9)	28 (8.1)	0.899
Previous MI [*n* (%)]	16 (3.5)	28 (8.1)	0.004
Diabetes [*n* (%)]	113 (24.8)	78 (22.7)	0.489
eGFR ≤60 ml/min/1.73 m^2^ [*n* (%)]	81 (17.8)	41 (11.9)	0.023
Peripheral vascular disease [*n* (%)]	10 (2.2)	12 (3.5)	0.267
Anemia [*n* (%)]	118 (25.9)	41 (11.9)	<0.001
Previous PCI [*n* (%)]	58 (12.7)	32 (9.3)	0.130
Previous CABG [*n* (%)]	4 (0.9)	2 (0.6)	0.631
Symptom to first medical contact, median (IQR), min	180 (117.8-401.5)	194 (82-389)	0.680
Killip class≥ II [*n* (%)]	69 (15.1)	58 (16.9)	0.508

Distributed continuous variables are expressed as the mean ± standard deviation; categorical variables are expressed as n (%). BMI, body mass index; LVEF, left ventricular ejection fraction; MI, myocardial infarction; eGFR, estimated glomerular filtration rate; PCI, percutaneous coronary intervention; CABG, coronary artery bypass graft; IQR, interquartile range.

**Table 2:** Treatment and procedural characteristics.

**Variable**	**Bivalirudin group (*n =* 456)**	**Heparin group (*n =* 344)**	***P*-value**
Door to wire (D2W), median (IQR), min	76 (60–120)	80 (56–112.5)	0.853
IRA [*n* (%)]			0.423
Left main	11 (2.4)	6 (1.7)	
Left anterior descending	250 (54.8)	188 (54.7)	
Left circumflex	60 (13.2)	58 (16.9)	
Right	135 (29.6)	92 (26.7)	
Multivessel disease [*n* (%)]	233 (51.1)	189 (54.9)	0.281
Arterial access [*n* (%)]			0.933
Radial artery	428 (93.9)	325 (94.5)	
Brachial artery	15 (3.3)	10 (2.9)	
Femoral artery	13 (2.9)	9 (2.6)	
Average stent number	1.6 ± 0.8	1.6 ± 0.9	0.986
Peri-procedural medication [*n* (%)]			
GPI	66 (14.5)	52 (15.1)	0.800
ACE inhibitor/ARB	263 (57.7)	200 (58.1)	0.895
β-blockers	270 (59.2)	215 (62.5)	0.346
Statins	413 (90.6)	307 (89.2)	0.536
Calcium blocker	71 (15.6)	49 (14.2)	0.603

Normally distributed continuous variables are expressed as the mean ± standard deviation; categorical variables are expressed as n (%). IQR, Interquartile range; IRA, infarction related artery; GPI, glycoprotein IIb/IIIa inhibitors; ACE, angiotensin-converting enzyme; ARB, angiotensin II receptor blocker.

### Post-PCI 30-day NACE

**Figure 2:**
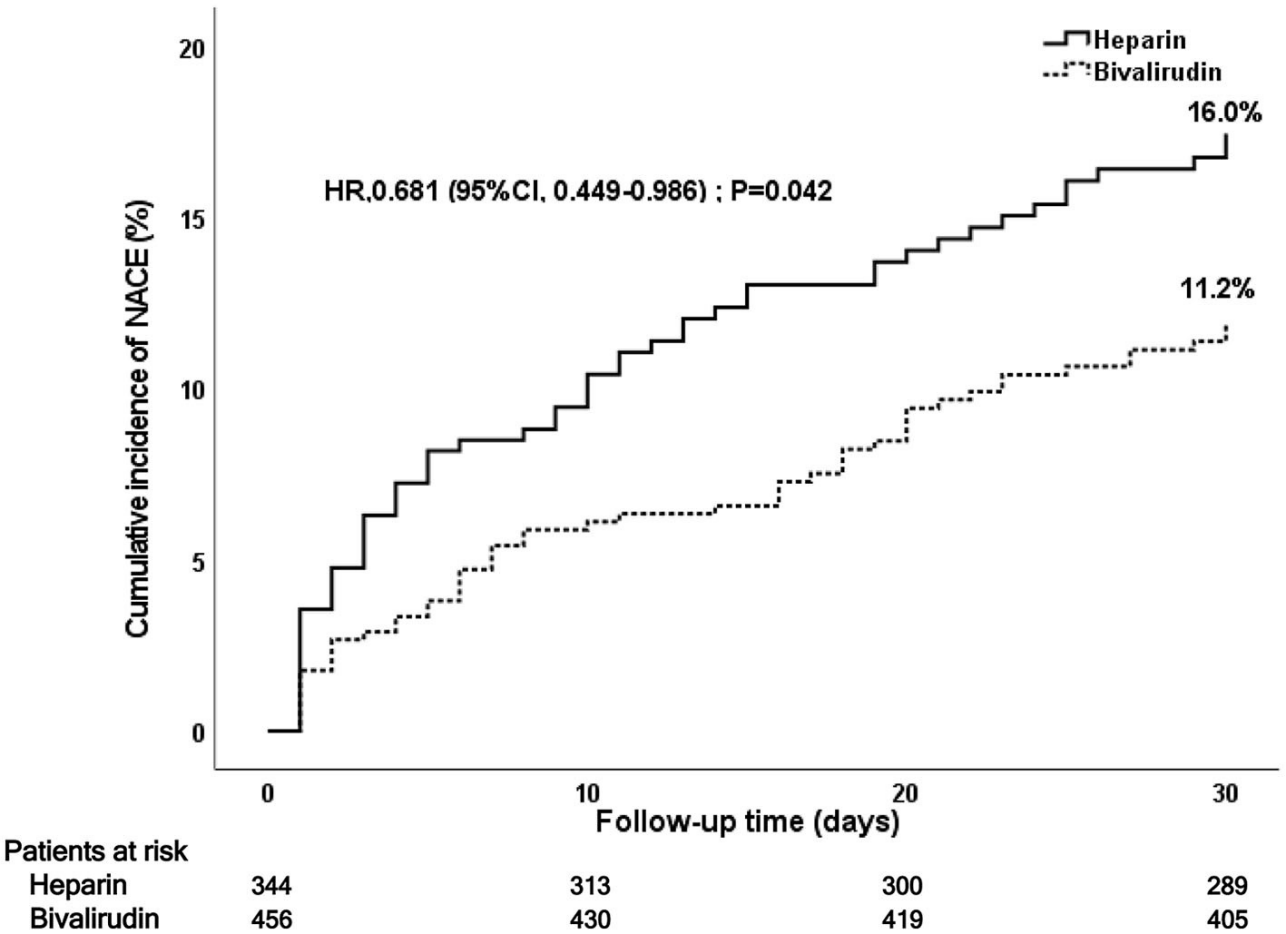
Kaplan–Meier curve of post-PCI 30-day NACE. PCI, percutaneous coronary intervention; NACE, net adverse clinical events.

During the 30-day follow-up after PCI, a total of 106 NACE (13.2%) occurred:52 NACE (11.2%) in the bivalirudin group and 51 NACE (16.0%) in the heparin group (see Figure 2 and Table 3). The difference was significant between the two groups (*P* = 0.042). Multivariate Cox regression analysis showed that the incidence of NACE was lower in the bivalirudin group than in the heparin group (*P* = 0.042). The composite of MACCE or bleeding requiring medical intervention (BARC 2–5 bleeding events) did not differ significantly between the groups (Table 3).

**Table 3:** Post-emergency PCI 30-day adverse events.

**Clinical event [*n* (%)]**	**Bivalirudin group (*n =* 456)**	**Heparin group (*n = 344*)**	**Crude**		**Adjusted**	
			**HR (95% CI)**	***P*-value**	**HR (95% CI)**	***P*-value**
NACE	55 (11.2)	51 (16.0)	0.676 (0.462–0.990)	0.042	0.665(0.449–0.986)	0.042
MACCE+ BARC 2-5	35 (7.7)	32 (9.3)	0.821 (0.508–1.326)	0.417	0.775 (0.473–1.270)	0.312
MACCE	23 (5.0)	19 (5.5)	0.912 (0.497–1.675)	0.767	0.845 (0.453–1.577)	0.597
Cardiac death	18 (4.0)	11 (3.2)	1.238 (0.585–2.620)	0.576	1.037 (0.480–2.240)	0.926
MI	2 (0.5)	3 (0.9)	0.507 (0.085–3.033)	0.448	0.472 (0.076–2.932)	0.420
Stroke	2 (0.5)	2 (0.6)	0.758 (0.107 −5.379)	1.781	1.064 (0.144–7.873)	0.952
TVR	3 (0.7)	4 (1.2)	0.567 (0.127–2.535)	0.452	0.584 (0.122–2.807)	0.502
Any bleeding	30 (6.7)	39 (11.5)	0.563 (0.350–0.906)	0.016	0.563 (0.345–0.920)	0.022
BARC 1	14 (3.2)	24 (7.1)	0.429 (0.222–0.830)	0.010	0.459 (0.233–0.901)	0.024
BARC2-5	16 (3.6)	15 (4.4)	0.804 (0.397–1.626)	0.542	0.755 (0.364–1.566)	0.450
BARC 2	10 (2.2)	12 (3.6)	0.629 (0.272–1.455)	0.274	0.629 (0.263–1.505)	0.297
BARC3-5	6 (1.3)	3 (0.9)	1.510 (0.378–6.037)	0.557	1.211 (0.295–4.975)	0.791
Stent thrombosis	1 (0.2)	1 (0.3)	0.762 (0.048–12.178)	0.847	0.987 (0.060–16.249)	0.993

Categorical variables are expressed as n (%). NACE, net adverse clinical events; MACCE major adverse cardiac and cerebrovascular events; MI, myocardial infarction; TVR, target vessel revascularization; BARC, Bleeding Academic Research Consortium.

### Post-PCI 30-day MACCE and stent thrombosis

**Figure 3:**
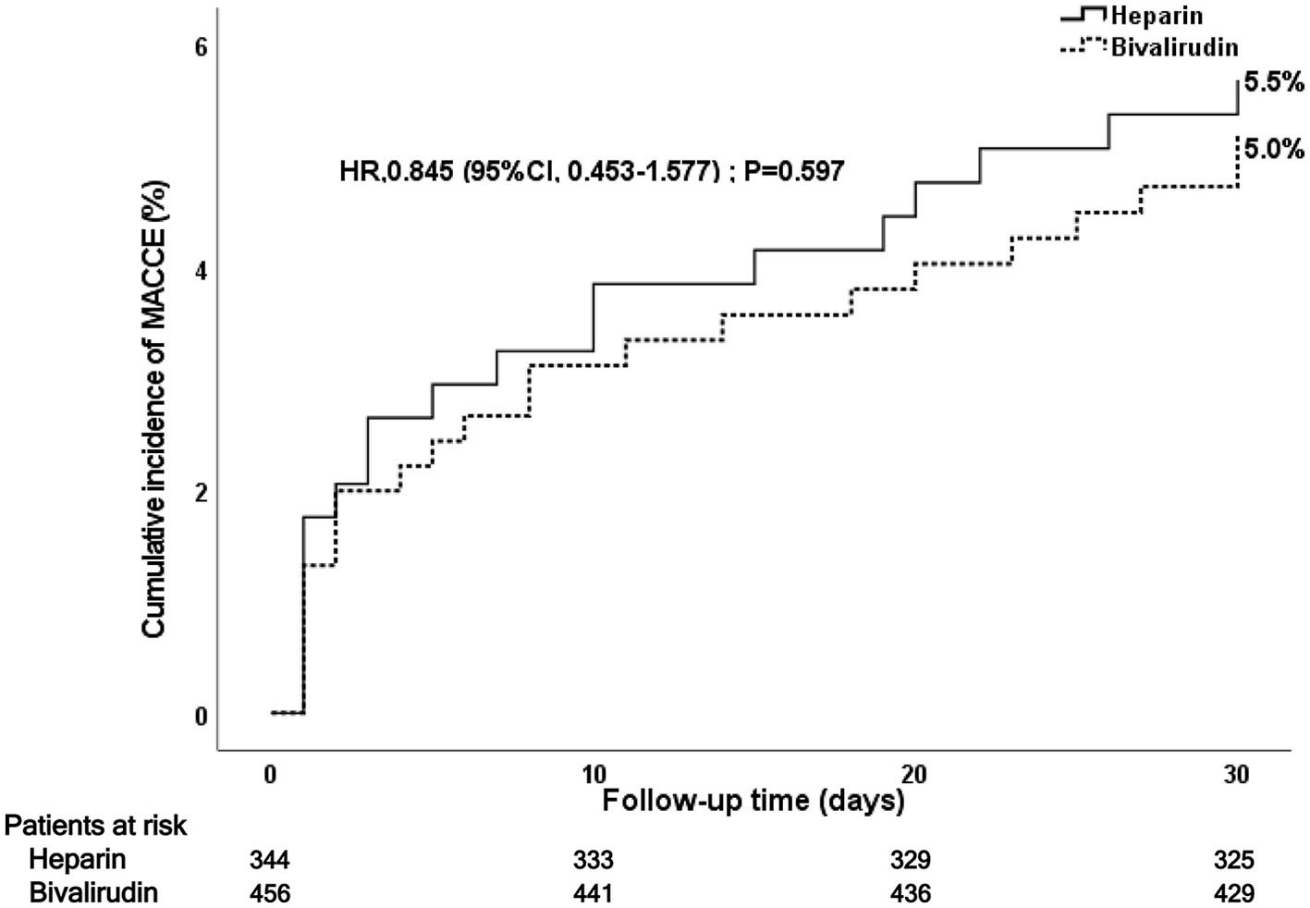
Kaplan–Meier curve of post-PCI 30-day MACCE. PCI, percutaneous coronary intervention; MACCE, major adverse cardiac and cerebrovascular events.

During the 30-day follow-up after PCI, a total of 42 MACCE occurred: 23 MACCE (5.0%) in the bivalirudin group and 19 MACCE (5.5%) in the heparin group (see Figure 3 and Table 3). The difference did not reach statistical significance (*P* = 0.767). Multivariate Cox regression analysis showed no significant between-group difference in the incidence of MACCE (*P* = 0.597). No significant difference was observed in cardiac death (4.0 vs. 3.2%), recurrent myocardial infarction (0.5 vs. 0.9%), stroke (0.5 vs. 0.6%), target vessel revascularization (0.7 vs. 1.2%), or stent thrombosis (0.2 vs. 0.3%) between the bivalirudin group and the heparin group (*P* > 0.05).

### Post-PCI 30-day bleeding events

**Figure 4:**
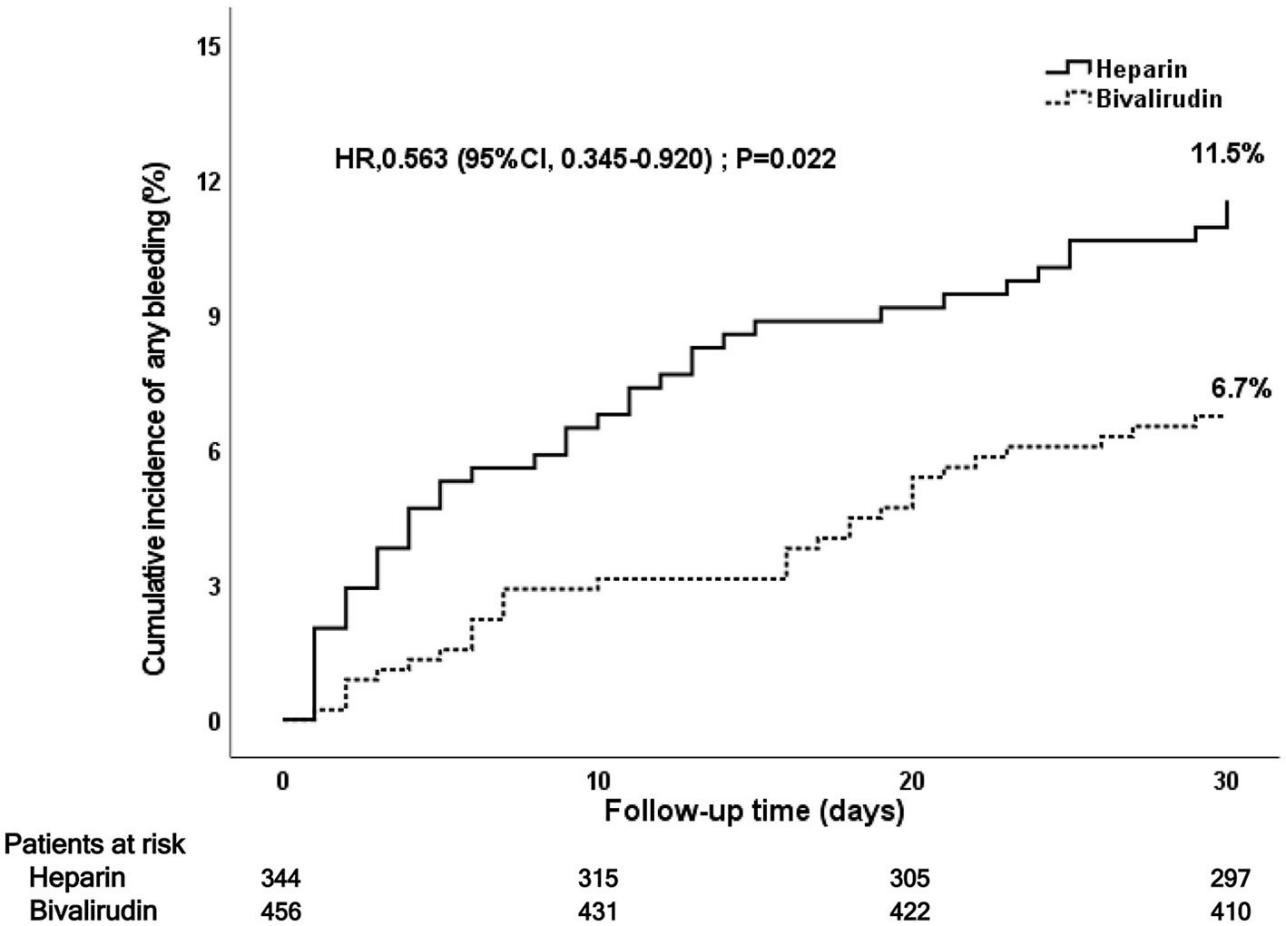
Kaplan–Meier curve of post-PCI 30-day bleeding events. PCI, percutaneous coronary intervention.

During the 30-day follow-up after PCI, bivalirudin was associated with a lower rate of bleeding events at 30 days (6.7 vs. 11.5%, *P* = 0.016) than UFH (see Figure 4 and Table 3). This significance was mainly a consequence of the reduction in BARC 1 bleeding events in the bivalirudin group compared to the UFH group (3.2 vs. 7.1%, *P* = 0.010). Multivariate Cox regression analysis also showed that the rates of all bleeding events and BARC1 bleeding events were significantly lower in the bivalirudin group than in the heparin group. No significant difference was observed in BARC 2–5 bleeding events (3.6 vs. 4.4%), BARC 2 bleeding events (2.2 vs. 3.6%), and BARC 3–5 bleeding events (1.3 vs. 0.9%) between the bivalirudin group and heparin group (*P* > 0.05).

## Discussion

In this prospective cohort, multicenter trial, we explored the effectiveness and safety of bivalirudin in patients with STEMI who were undergoing primary PCI and receiving treatment with aspirin and ticagrelor. The main findings were as follows: (1) bivalirudin was associated with a significantly lower rate of NACE than heparin at 30 days after PCI, which was mainly derived from a significant reduction of minimal bleeding, but not major bleeding. (2) Bivalirudin therapy showed a similar risk of causing ischemic events, including ST, compared to heparin.

Administration of the optimal peri-procedural antithrombotic regimen during PCI is essential to balance the risk of bleeding and ischemia. In previous studies comparing bivalirudin with heparin, including the HORIZONS-AMI (10) and BRIGHT (12) trials, bivalirudin has been associated with reduced bleeding but increased early stent thrombosis, a risk that can be mitigated by a prolonged infusion. It is noteworthy that clopidogrel was the default P2Y12 inhibitor used in those trials instead of ticagrelor or prasugrel. Potent P2Y12 inhibitors, such as ticagrelor, have been strongly recommended over clopidogrel to reduce the risk of ischemic recurrences (4). The effective ischemic protection offered by stronger P2Y12 inhibition is potentially counterbalanced by an increased risk of bleeding (4, 18). At variance, the multicenter VALIDATE-SWEDEHEART trial (19), which enrolled patients with STEMI undergoing urgent PCI with potent P2Y12 inhibitors (about 81% of the patients received ticagrelor) and without planned GPI, found similar cardiovascular outcomes and bleeding risk between groups. Moreover, in the VALIDATE-SWEDEHEART trial (19), the rate of stent thrombosis was lower in the bivalirudin group—a finding that might be attributed to the prolonged bivalirudin infusion. Notably, around one-third of patients in the bivalirudin group were given heparin right before the procedure, which introduces imbalances in treatment effects. Thus, even after VALIDATE-SWEDEHEART trial (19), there is no definitive answer to the question of whether to use bivalirudin or heparin during PCI in the current era (8).

Our study has represented contemporary PCI practice (e.g., predominantly performed *via* radial access, use of potent P2Y12 inhibitor, and limited use of GPI), which made our findings more valid. Both before and after correcting the baseline of the two groups, we found that peri-procedural use of bivalirudin during PCI in patients with STEMI treated with potent P2Y12 inhibitors showed a lower incidence of NACE events compared with heparin, mainly due to the reduced risk of minimal bleeding events (BARC1 bleeding events). The risk for BARC 2 bleeding events in the bivalirudin group tended to be lower (10 [2.2%] vs. 12 [3.6%]; HR: 0.629, *P* = 0.297); however, the difference did not reach statistical significance. Given the low absolute number of BARC 2 bleeding events at 30-day follow-up, our study may not be powered enough to detect significant differences in BARC 2 bleeding events at 30 days.

Our results demonstrated that the anti-ischemic effect of bivalirudin was similar to that of heparin in patients with STEMI who underwent primary PCI. At 30 days, stent thrombosis occurred in 0.2% receiving bivalirudin and 0.3% receiving heparin (*P* = 0.847). The relatively low rates of stent thrombosis overall compared with previous similar studies (10, 20) and the absence of a significant between-group difference in ischemic event rates might be due to the treatment with ticagrelor and prolonged infusion of bivalirudin after primary PCI. Ticagrelor has shown evidence of a lower incidence of adverse ischemic events and stent thrombosis compared with clopidogrel in several trials (4, 21). A recently published meta-analysis (22) including four trials on bivalirudin therapy in patients undergoing primary PCI has suggested that bivalirudin, with the high-dose delayed application method after PCI, was associated with a lower risk of early definite stent thrombosis compared with treatment with heparin.

This study has some limitations. First, anticoagulant regimen selection was not randomized and was at the discretion of the treating physician, which makes all comparisons subject to potential biases underlying the choice of therapy. Despite multiple adjustments for differences in baseline characteristics between treatment groups, unmeasured confounders influencing outcomes cannot be excluded. Randomized controlled trials are needed to validate the results. Second, the information on the use of other medications during the 12-month follow-up period was not available, which has potential implications for clinical outcomes in the different cohorts. Some evidence suggests that bleeding affects long-term mortality (6). Third, given the low absolute number of events at 30-day follow-up, our study was not powered enough to detect significant differences in stroke, MI, TVR, stent thrombosis, BARC 2 bleeding events, and BARC 3–5 bleeding events.

## Conclusion

In patients with STEMI who were undergoing primary PCI and receiving treatment with aspirin and ticagrelor, bivalirudin was associated with decreased rates in NACE and minimal bleeding events without significant differences in the rates of MACCE or stent thrombosis when compared with heparin. Nevertheless, large randomized trials are warranted to confirm these observations.

## Data availability statement

The raw data supporting the conclusions of this article will be made available by the authors, without undue reservation.

## Ethics statement

The studies involving human participants were reviewed and approved by the Clinical Research Ethics Committee of The First Affiliated Hospital of University of Science and Technology of China. The patients/participants provided their written informed consent to participate in this study.

## Author contributions

X-FY and L-KM conceived and designed the study. H-WC, Q-ZX, JX, X-HZ, B-BL, and B-LX were involved in data collection, interpretation, and analysis. X-FY wrote the manuscript. L-KM was involved in the editing of the manuscript. All authors have read and approved the final manuscript.

## Funding

This study was supported by the Open Project of Anhui Provincial Cardiovascular Institute (Grant No. KF2018007) and the National Natural Science Foundation of China (Grant No. 82170263).

## Conflict of interest

The authors declare that the research was conducted in the absence of any commercial or financial relationships that could be construed as a potential conflict of interest.

## Publisher's note

All claims expressed in this article are solely those of the authors and do not necessarily represent those of their affiliated organizations, or those of the publisher, the editors and the reviewers. Any product that may be evaluated in this article, or claim that may be made by its manufacturer, is not guaranteed or endorsed by the publisher.
