# Supplementary figures and images for: Bivalirudin vs. heparin on a background of ticagrelor and aspirin in patients with ST-segment elevation myocardial infarction undergoing primary percutaneous coronary intervention: A multicenter prospective cohort study
Source: Front Cardiovasc Med. 2022 Oct 28;9:932054. doi: 10.3389/fcvm.2022.932054 (PMC9649932; doi:10.3389/fcvm.2022.932054)

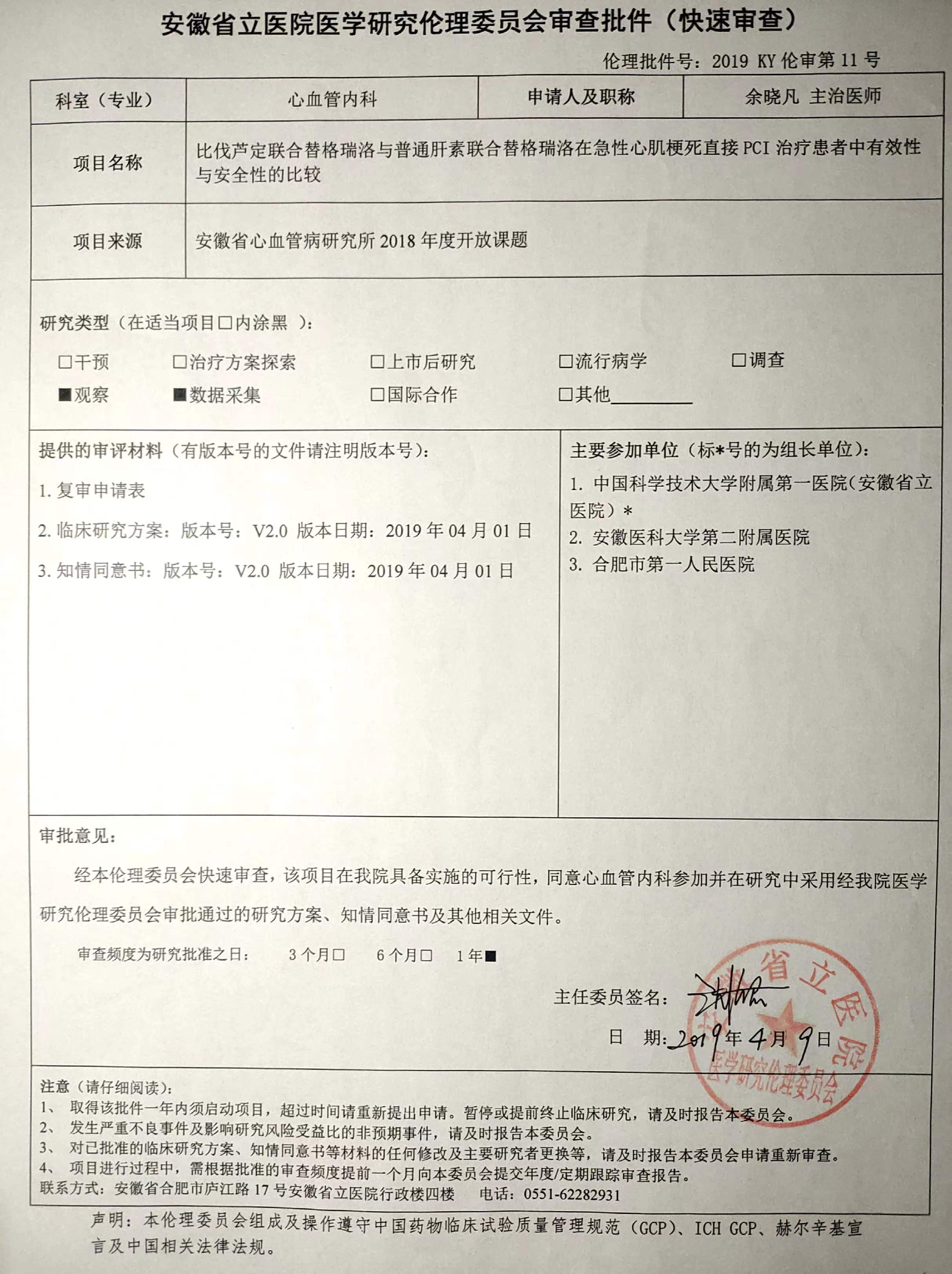

Supplement: Supplementary file 1 [file Image_1.JPEG]

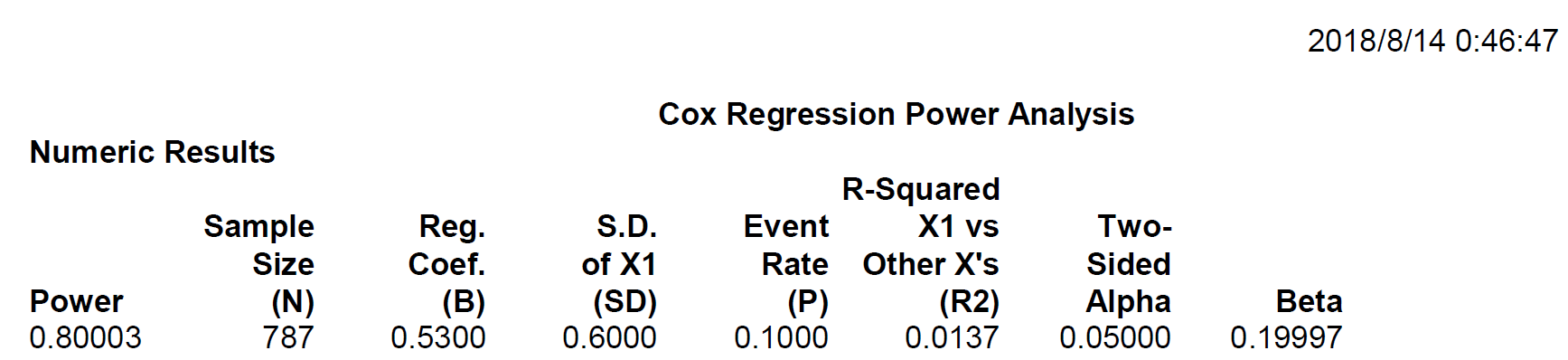

Supplement: Supplementary file 2 [file Image_2.TIF]
